# The potential role of AI in research priority setting exercises

**DOI:** 10.7189/jogh.15.03019

**Published:** 2025-06-02

**Authors:** John Garry, Mark Tomlinson, Maria Lohan

**Affiliations:** 1 Queen’s University Belfast, Department of Politics and International Relations, Northern Ireland, UK; 2 Stellenbosch University, Institute for Life Course Health Research, Cape Town, South Africa; 3 Queen’s University Belfast, School of Nursing and Midwifery, Northern Ireland, UK; 4 Hitotsubashi University, Hitotsubashi Institute for Advanced Studies, Tokyo, Japan

## Abstract

To help achieve the goals of accountability and research excellence, funding organisations often utilise evidence from research priority setting exercises (RPSEs), which distil, from data gathered from relevant stakeholders, a systematic and ‘objective’ rank-order of research priorities. RPSEs are, however, costly and labour-intensive. Also, critics of RPSEs have highlighted certain limitations: insufficient representation of difficult-to-reach stakeholders, especially in low- and middle-income countries; a lack of genuine stakeholder engagement; wide variation in the extent to which exercises are documented; a lack of specificity in the identified priorities; and minimal impact of the priorities. Artificial intelligence (AI) tools such as ChatGPT may potentially help, valuably complementing conventional RPSEs. While the opacity of AI decision-making is a limitation, advantages include speed, affordability, and highly inclusive distillation of the vastness of existing human knowledge. We encourage research identifying the extent to which AI can replicate conventional RPSEs. We suggest that AI tools could complement conventional approaches either at the initial question generation stage or in generating supplementary insights for reflection at the data analysis stage. Also, under conditions of high existing stakeholder engagement and an extant prevalence of conventional RPSEs, AI-only studies may be valuable.

Funding organisations typically wish to achieve a number of goals when making decisions about how to allocate their scarce resources. When deciding which particular research questions to prioritise, they need their decision-making process to be transparent and systematic in order to offset any possible suspicion of arbitrariness or unconscious favouritism. They also want the questions they prioritise to lead to the most valuable and fruitful research.

## RESEARCH PRIORITY SETTING EXERCISES

To maximise accountability and research excellence, funding organisations often draw on the results of research priority setting exercises (RPSEs) [1]. The most well-known RPSEs use the Delphi methodology [2] or follow the approach of the Child Health and Nutrition Research Initiative (CHNRI) [3]. While methods may vary somewhat in design, RPSEs broadly follow three steps:

1. Identify relevant stakeholders. These are often a combination of relevant academic experts and practitioners.
2. Identify, *via* engagement with stakeholders, a list of possible research priorities.
3. Subsequently, identify, *via* engagement with stakeholders, how the stakeholders rate the importance of each possible priority on a number of criteria (such as the likely impact of addressing certain research questions/sub-themes and the feasibility of actually addressing them), and derive from these ratings an overall ranking of the research priorities.

The engagement with stakeholders is either through in-person consensus building (Delphi) or the remote quantitative ranking of options (CHNRI and increasingly Delphi exercises). The generated rank-ordered listing of possible research priorities may then be perused by the funder. The list provides a solid sense of what experts in the field think should be prioritised. Importantly, the list is not deterministic: funders do not have to base funding calls on the top-ranked research themes. Rather, the list serves as a map that enables decision-makers to systematically navigate challenging spending terrain. For instance, a research sub-theme/question ranked in 9th place may be attractive because the experts have identified it as moderately impactful and highly feasible, and also because the sub-theme relates to a particular part global region the funder is keen to focus on.

When the time comes for the line manager of the decision-maker to reflect on the year’s spend and ask for a compelling account of why certain themes rather than others were prioritised, the RPSE listing can form part of a transparent and systematic account of the decision-maker’s prioritisation process. The quality of the identified research priorities – in terms of impact, feasibility and other relevant criteria – may be defended because the RPSE utilised existing expert knowledge in a thoughtful way to carefully select high-quality research priorities. The increasing number of RPSEs that have been conducted, typically – though not exclusively – in the area of health-related research [4,5] suggests that they do indeed serve a useful role both regarding accountability and high-quality generation of valuable priorities.

## LIMITATIONS

There is, however, a substantial literature critiquing research priority setting processes [6-8]. In a review of health research priority setting exercises in eight countries, Tomlinson and colleagues [8] found a lack of genuine stakeholder engagement; wide variation in the extent to which exercises were documented; a lack of specificity in the identified priorities, which often, for example, referred to a broad disease category rather than a specific question; and the absence of a mechanism for findings to be publicized, appealed and contested. Perhaps the most oft-cited criticism has been that RPSEs have not been seen as legitimate and fair, as decisions were often perceived as being driven by individual bias – the most powerful person in the room having inordinate power [7]. Recent new methodologies such as CHNRI have attempted to respond to some of these critiques by engaging a more diverse group of stakeholders and making the exercises more legitimate and fairer [9] through a process of systematic and transparent documentation [10,11]. Even with the best of intentions, however, current methods continue to struggle in important ways: with appropriate representation of difficult-to-reach stakeholders; low response rates from low- and middle-income countries; ensuring that priorities are revised; and finally, and perhaps most importantly, a process to track whether in fact established priorities actually result in funding and/or research shifts on the ground [12].

## CAN AI HELP TO COMPLEMENT RPSEs?

With these criticisms of RPSEs in mind, plus the expensive and time-consuming nature of the exercises, does the recent proliferation of the use of AI tools such as ChatGPT present a novel challenge? Are conventional RPSEs vulnerable to AI disruption?

A recent study used ChatGPT to generate a priority listing on the subject of ageing, in which the authors argued that the results appeared compelling, but had to be closely interpreted by humans [13]. In another study, researchers found that ChatGPT can produce results (a rank-order of research priorities) that are remarkably similar to those produced by a full-scale conventional RPSE empirical project that took months to complete [14]. The results generated by ChatGPT were broader, or more abstract, than those produced by the conventional RPSE, but the authors found the overall similarity of the two sets of findings remarkable [14].

These studies prompt two questions: should AI play a role in complementing conventional RPSEs? If so, how?

### Should AI play a role? Theoretical response

‘I can see that it works in practice, but does it work in theory?’ [15], the late Irish *Taoiseach* (prime minister) and economist Garret Fitzgerald once asked in relation to a policy proposal. This is a pertinent question to ask of the ChatGPT ‘success’ in replicating an RPSE. What is actually going on inside ChatGPT? What theoretical account of how it came up with the priorities can be specified? 

To address these questions, let us reflect on steps 1–3 above in a conventional RPSE. Let us explicate the normative basis of each of the three steps and examine if a similarly persuasive normative account of the three steps can be elaborated regarding ChatGPT use as a short-cut complementary mechanism.

### Identify relevant stakeholders

Expertise is the normative defence of this first step in a conventional RPSE [3]. Only academics and practitioners who are deemed by the RPSE organisers to have valuable knowledge and experience are included. In contrast, ChatGPT does not use transparent inclusion and exclusion criteria. It essentially regards as ‘expert’ all textual materials that are on the internet that relate to the topic in question. Equally, ChatGPT explicitly excludes all humans and materials, no matter how expert, that do not have an internet presence.

An advantage of ChatGPT is that it may capture a much wider array of stakeholders than conventional RPSEs. By aggregating a vast range of expert opinions from the internet, it might capture expertise in a way that traditional RPSEs cannot, and may also potentially capture non-expert opinion.

### Identify a list of possible research priorities

In conventional RPSEs, the organisers highlight the importance of being inclusive rather than exclusive in the compilation of an initial set of potential sub-themes/research questions. ChatGPT may also achieve such inclusion because it incorporates everything ever written on the topic that is on the internet.

### Ranking the research priorities

Replicability and precision are among the normative bases of this step in conventional RPSEs. Using transparent and replicable surveys, and using transparent and replicable statistical code to analyse the data, means that it is clear how the rank-order of priorities has been generated, allowing the process to be repeated by other researchers. 

In contrast, because ChaptGPT is a black box into which we cannot clearly peer, it is not possible to tell a story about what exactly ChatGPT ‘is doing. What we do know is that, in at least one instance, it can broadly replicate a conventional RPSE. Only knowing this much is theoretically superficial, however. It is a bit like listening to someone confidently predicting that the sun will rise every morning. The prediction is accurate, but a far cry from a general model of the solar system that will enable us to actually understand why the sun rises every morning. In possible defence of AI tools such as ChatGPT, it may be argued that even though the details of the mechanism used are unclear, the broad approach is clear in that it interrogates the vastness of stored human collective knowledge on a topic which conventional RPSEs cannot do as they must identify some knowledge holders who are willing to take part in the exercise. Also, the AI-generated results are replicable in the sense that repeating the request is likely to produce highly similar results.

Further normative bases of this third step are the intrinsic value of RPSEs in fostering deliberation [11] and the considered emergence of the collective view of expert stakeholders. This is linked to a further normative advantage, which is engagement in the process, which, in turn, leads to buy-in and a more enthusiastic commitment to efficiently put into effect the policy consequences of the prioritisation process. In contrast, if AI is used, the absence of deliberation and engagement could lead to the produced priority listing being perceived as coming out of nowhere, which would be alienating for relevant stakeholders. However, in defence of AI, in circumstances where networked engagement already exists, stakeholders may be assured that the AI-produced priorities are essentially a neat summary of existing human knowledge. 

In terms of steps 1–3, what ChatGPT essentially does is conduct all three steps simultaneously rather than, as in conventional RPSEs, distinctly and sequentially. In one single fell swoop in a matter of seconds, ChatGPT distils from all internet stakeholders (texts, not humans) a list of research priorities that is in rank-order format. What would Garret Fitzgerald make of all this? Yes, it works, but we do not really know why. The opacity of AI decision-making may well present a fundamental epistemic challenge, not just a lack of public understanding. But this may be offset by the advantages of speed, affordability, and highly inclusive distillation of the vastness of existing human knowledge.

### Should AI play a role? Empirical response

We may treat the ChatGPT approach in the same way we would treat any ‘new shortcut’ methodology that claims it can reliably replicate an existing methodology [16]. Specifically, let us test its replication-based validity much more firmly, and on many more cases. The first way that this could be done is to take a set of recent RPSEs on a particular topic, generate ChatGPT results on the same topic, and compare the two sets of findings. The greater the number of instances in which the ‘new’ approach can broadly replicate the old approach, the more confidence we may have. 

We do not demand complete replication because conventional RPSEs are imperfect, and what they miss may, to some extent, be picked up by the new AI method. However, we would at least expect a significant level of replication. Note that in this replication exercise, the request for AI would have to be limited to using evidence from a time period prior to the publication of the RPSE it is seeking to replicate. This caveat does not apply if the exercise with the two approaches is done in parallel. 

This type of exercise could also identify the conditions under which ChatGPT can most accurately replicate the existing RPSE gold standard. Perhaps on topics that have been well debated rather than new and niche topics? Perhaps on topics that are more broad brush than nuts and bolts? Perhaps much more so in some languages than others? This type of research could amend the ‘ChatGPT or RPSE?’ question to ‘Under what conditions, if any, is it reasonable to use ChatGPT rather than RPSE?’

A second programme of empirical research could engage ChatGPT in new RPSEs in a way that will help us understand the potential value of ChatGPT. Imagine we set about conducting a conventional RPSE and, following step 1, identified a relevant set of human experts. Imagine also, we distinctly conducted a ChatGPT exercise and generated a rank-ordered set of 10 priorities on the relevant theme. Next, we present a randomised ordered version of the ChatGPT-generated 10 priorities to our set of human experts, but do not tell them the priorities are ChatGPT-generated. As per a conventional RPSE, we ask our human experts to rate (or directly rank-order) the list of priorities. We also ask our experts to judge the reasonableness of the set of included priorities. 

Empirically, we compare the ChatGPT and human rank-ordering of the 10 priorities. Our first test relates to similarity of rank-ordering across the two approaches, with greater similarity prompting greater confidence that a set of priorities can indeed be rank-ordered by ChatGPT in a way that is similar to humans. Our second test relates to responses to the questions on the reasonableness of inclusion, from which we can establish humans’ perception of the substantive validity of the ChatGPT-generated priorities. In a version of the Turing test, do the ‘experts’ in a conventional RPSE flinch at the ChatGPT-generated list of priorities? Or do they accept them just as human experts accept the offered priorities in RPSEs?

### How exactly can AI play a role?

Once it has been established that AI does at least a reasonable job of replicating the existing method, we can focus on identifying exactly how AI can complement conventional RPSEs. 

Priorities generated by AI could play a role at the question generation stage of a conventional RPSE. They could contribute to a ‘long list’ of possible plausible questions, derived from a range of sources, from which the RPSE organisers choose a selection to include in the survey phase of the exercise. Including the AI-generated priorities as one component at this stage could lessen the risk that important questions were not excluded for consideration on the long list. 

At the final data analysis stage of a conventional RPSE, AI could augment the conventional analysis by asking supplementary questions. For example, AI could be asked to generate priorities that are especially important for particular audiences: for instance, academics and practitioners [13]. It could also produce priorities especially relevant for low- or high-income countries, or especially relevant for males or females. Or AI could identify priorities that vary by important criteria: for instance, priorities that are particularly easy to implement or priorities that are particularly impactful. The output of such requests could be reflected upon in the context of reflecting on the results of the conventional RPSE. 

There may be instances where an AI-generated set of priorities could be valuable on its own, without the need for any directly associated conventional RPSE: perhaps when a number of RPSEs have recently been conducted and there is a high existing level of stakeholder engagement. Under such conditions, the risk of stakeholders feeling alienated by the AI-only process may be minimised. Also, if the results are examined in the context of the recent set of human-conducted exercises, concerns over the lack of human oversight may be assuaged.

## CONCLUSIONS

AI-based RPSEs and conventional RPSEs may be mutually supportive and complementary rather than antagonistic. We encourage further studies to grapple with how AI can help conventional approaches and help funders achieve their twin aims of accountable decisions and enabling the identification of excellent research priorities.
